# Fluorescence-Guided Surgery

**DOI:** 10.3389/fonc.2017.00314

**Published:** 2017-12-22

**Authors:** Tadanobu Nagaya, Yu A. Nakamura, Peter L. Choyke, Hisataka Kobayashi

**Affiliations:** ^1^Molecular Imaging Program, Center for Cancer Research, National Cancer Institute, National Institutes of Health, Bethesda, MD, United States

**Keywords:** fluorescence-guided surgery, activatable probe, monoclonal antibodies, molecular imaging, always-on probe

## Abstract

Surgical resection of cancer remains an important treatment modality. Despite advances in preoperative imaging, surgery itself is primarily guided by the surgeon’s ability to locate pathology with conventional white light imaging. Fluorescence-guided surgery (FGS) can be used to define tumor location and margins during the procedure. Intraoperative visualization of tumors may not only allow more complete resections but also improve safety by avoiding unnecessary damage to normal tissue which can also reduce operative time and decrease the need for second-look surgeries. A number of new FGS imaging probes have recently been developed, complementing a small but useful number of existing probes. In this review, we describe current and new fluorescent probes that may assist FGS.

## Introduction

Surgery is a primary mode of treatment for many malignancies. For example, 63–98% of patients with lung, breast, bladder, and colorectal cancer will undergo surgery (1). The goal of surgery is to safely remove as much cancer as possible. The degree to which cancer is removed relates closely to prognosis. However, the ability to resect tumor currently relies on the visual localization of the tumor and/or the ability to palpate it. The former is limited by the low contrast between tumors and background tissue and many small tumors may be missed. Moreover, the determination of tumor margins must often be done blindly followed by frozen section pathologic analysis.

The presence of residual tumor cells after resection is considered a strong predictor of tumor recurrence and, therefore, survival. Many studies show that positive margins, defined as the identification of tumor cells at the cut edge of a surgical specimen, are associated with increased local recurrence and indicate a poor prognosis in most cancer types including head and neck cancers (2), breast cancer (3, 4), non-small-cell lung cancer (5), colorectal cancer (6), bladder cancer (7), and prostate cancer (8). Despite advances in preoperative imaging such as computerized tomography (CT), magnetic resonance imaging (MRI), and positron emission tomography, surgical margin positivity rate has not changed significantly over the past several decades (9), with margin positivity rates of 15–60% across all cancers (10–16). Currently, the standard of care for achieving negative margins rests on visual inspection, palpation, and intraoperative histopathological analysis of frozen tumor margins all of which have severe limitations. The naked eye is limited in its ability to detect small tumors. Palpation is limited in sensitivity and is increasingly not used due to the increased utilization of robotic laparoscopic surgery. Intraoperative frozen section analysis is limited to certain tissue types, is time-consuming, and is prone to sampling error. Frozen section analysis is discrepant with permanent pathology in 5–15% of cases (17).

A number of non-optical imaging methods have been proposed during surgery. Typically, these methods are not targeted to the tumor *per se* but rely on anatomic abnormalities to define the tumor. For instance, intraoperative CT and MRI have played a significant role in the field of neurosurgical image guidance (18–20). However, intraoperative systems are costly, complex and require space. Moreover, their use interrupts the normal workflow of the surgical procedure lengthening operative/anesthesia times. These methods are, mainly used for neurosurgery at major medical centers.

Therefore, practical methods for augmenting the surgeon’s ability to resect tumors are needed. One such method is fluorescence-guided surgery (FGS). The first use of fluorescence imaging in surgery dates back to 1948 when surgeons used intravenous fluorescein to enhance intracranial neoplasms during neurosurgery (21). Since then, additional fluorescent agents have been used for a variety of surgical applications (22–24). Intraoperative fluorescence imaging offers the benefits of high contrast and sensitivity, low cost, absence of ionizing radiation, ease of use, safety, and high specificity (25, 26). Compared with standard unaided vision using white light imaging, real-time fluorescence imaging is helpful in identifying cancerous tissue and delineating tumor margins. Moreover, improved visualization of the cancer can reduce damage to important normal structures such as nerves, blood vessels, ureters, and bile ducts.

In this review article, we focus on the currently used Food and Drug Administration (FDA)-approved fluorescent probes and new types of fluorescence imaging probes for FGS that are under development.

## Current FGS

The exponential growth in the field of FGS is demonstrated by the number of published articles in the field, which has grown from under 50/year in 1995, to nearly 500/year in 2015 (27). Furthermore, FGS has enjoyed a number of preliminary successes (23, 28) and some FGS techniques have already achieved clinical success (29). FGS may improve tumor resection rates while minimizing normal tissue resection (9, 30, 31). This can translate into improved clinical outcomes.

Compared to expensive traditional imaging methods, optical methods are less costly and require less space. One cost estimate of the fluorescence-assisted resection and exploration (FLARE) system is 120,000 USD and 40,000 USD for the mini-FLARE (32, 33). Of course, this does not include the cost of the optical probe itself but the overall costs are much lower than with conventional imaging. Moreover, because it is portable a single instrument could be shared among multiple operating rooms.

Fluorescence-guided surgery has been currently used for multiple surgical situations, including sentinel lymph node (SLN) mapping, identification of solid tumors, lymphography, angiography, and anatomical imaging during surgery. Importantly, FGS can be used seamlessly during the procedure without interrupting the surgeon’s workflow. This integrates FGS into the surgery creating numerous opportunities for its use. We summarize current clinical and preclinical FGS techniques in Table 1.

**Table 1:** Current clinical and preclinical fluorescence-guided surgery techniques.

Application	Types	Contrast agent	Status
Sentinel lymph node mapping	Breast cancer	Indocyanine green (ICG) (34–37)	Clinical
		Methylene blue (MB) (38, 39)	Clinical
	Melanoma	ICG (40, 41)	Clinical
	Head and neck cancer	ICG (42)	Clinical
	Lung cancer	ICG (43)	Clinical
	Esophagus cancer	ICG (44, 45)	Clinical
	Gastric cancer	ICG (46, 47)	Clinical
	Colorectal cancer	ICG (48)	Clinical
	Anal cancer	ICG (49)	Clinical
	Prostate cancer	ICG (50–52)	Clinical
	Penile cancer	ICG (51, 52)	Clinical
Lymphography	Lymph flow	ICG (53–55)	Clinical
Angiography	Cerebral aneurysm	Fluorescein sodium (56–58)	Clinical
	Coronary artery bypass grafting	ICG (59, 60)	Clinical
	Abdominal aortic aneurysm	ICG (61)	Clinical
	Abdominal surgery	ICG (62, 63)	Clinical
	Reconstructive surgery	ICG (64–70)	Clinical
Anatomic imaging	Cholangiography	ICG (71, 72)	Clinical
	Pancreas	MB (73)	Preclinical
		T700-F (74)	Preclinical
	Ureters	MB (75)	Preclinical
	Nerves	Various fluorescently labeled peptide (NP) (76, 77)	Preclinical
	Parathyroid and thyroid grands	T700 and T800 fluorophores (78)	Preclinical
	Endocrine grands	Various near-infrared fluorophores (79–81)	Preclinical
Tumor imaging	Malignant glioma	5-ALA (82–86)	Clinical
		Fluorescein sodium (87–89)	Clinical
		BLZ-100 (90)	Clinical
		GB119 (91)	Preclinical
	Brain metastases	Fluorescein sodium (92, 93)	Clinical
	Head and neck cancer	IRDye800CW conjugate (94, 95)	Clinical
		IRDye700DX conjugate (96)	Clinical
	Hepatocellular carcinoma	ICG (97–100)	Clinical
	Liver metastases	ICG (99)	Clinical
	Breast cancer	MB (101)	Clinical
		EC17 (102)	Clinical
		IRDye800CW conjugate (102)	Clinical
		LUM015 (103)	Clinical
		AVB-620 (104)	Clinical
	Lung and chest masses	ICG (105)	Clinical
		Folate-fluorescein isothiocyanate (FITC) (106)	Clinical
		EC17 (107)	Clinical
		OTL38 (108)	Clinical
	Ovarian cancer	ICG (109)	Clinical
		Folate-FITC (28)	Clinical
		EC17 (110)	Clinical
		OTL38 (111)	Clinical
		gGlu-HMRG (112)	Preclinical
	Pancreatic cancer	Green fluorophore conjugate (113, 114)	Preclinical
		IRDye800CW conjugate (102)	Preclinical
	Insulinoma	MB (73)	Preclinical
	Solitary fibrous tumor (pancreas)	MB (115)	Preclinical
	Renal cell carcinoma	EC17 (116)	Clinical
		OTL38 (102)	Clinical
	Bladder cancer	5-ALA/HAL (117–120)	Clinical
	Prostate cancer	ICG conjugate (121)	Preclinical
		5-ALA (122)	Clinical
	Gastric cancer	ICG (123–125)	Clinical
	Colorectal cancer	Green fluorophore conjugate (113)	Preclinical
		IRDye800CW conjugate (126)	Clinical
		gGlu-HMRG (127)	Preclinical
	Basal cell carcinoma	5-ALA (128)	Clinical
		GB119 (129)	Preclinical
	Sarcoma	LUM015 (103)	Clinical
	Parathyroid adenoma	MB (130)	Clinical
Laparoscopic- and robotic-assisted surgeries	Nephrectomy	ICG (131)	Clinical
	Cholecystectomy	ICG (72, 132)	Clinical
	Esophagectomy	ICG (133)	Clinical
	Gastrectomy	ICG (134)	Clinical
	Adrenalectomy	ICG (135, 136)	Clinical
Fluorescence endoscopy	Brain aneurysm	ICG (137–139)	Clinical
	Endonasal surgery	ICG (140–142)	Clinical
	Angiography	ICG (142, 143)	Clinical
	Brain tumor	ICG (140, 144, 145)	Clinical
	Head and Neck tumor	ICG (146)	Clinical
	Gastric cancer	ICG (123–125)	Clinical
Marking tumor	Colonic tattooing	ICG (147–149)	Clinical

## Clinically Available Fluorescence Imaging

There has been an explosion of interest in FGS, which has led to a steady demand for new fluorescence imaging devices and probes. Currently, most FGS imaging has been performed with the Novadaq SPY system which was the first to be approved by FDA in 2005; however, several new fluorescence imaging systems have subsequently been approved by the FDA as shown in Table 2. These systems are approved for a variety of procedures including imaging blood flow, tissue perfusion, and circulation in free flaps, plastic surgery, and reconstructive surgery. These systems are portable making their positioning within a room completely customizeable to the situation. Hand-held cameras of PDE and Fluobeam, for instance, possess the advantage of being compact and convenient for real-time fluorescence imaging. Other cameras such as Quest Spectrum and VS3 Iridium simultaneously show the white light image and the fluorescent probe image overlay which reduces distractions for the surgeon (150, 151). In the field of breast oncology, the SPY system has been applied to monitor skin perfusion in nipple-sparing mastectomies using ICG as the imaging probe. This method can guide the location of mastectomy incisions and minimize ischemic complications (152).

**Table 2:** Clinically available Food and Drug Administration-approved fluorescence imaging systems.

Imaging system	Company	Excitation wavelength(s) (nm)	Light source	Working distance (cm)	Field of view (cm)	Real-time overlay
SPY	Novadaq Technologies	805	Laser	~30	19 × 14	No
PDE	Hamamatsu Photonics	760	LED	~20	5 × 5 to 10 × 6.7	No
Fluobeam 700 (800)	Fluoptics Minatec	680 (750)	Laser	15 ~ 25	2.2 × 1.5 to 20 × 14	No
Quest Spectrum	Quest Medical Imaging	400–1,000	Laser	5~	2.25 × 2.25 (5 cm distance)	Yes
VS3 Iridium system	VisionSense	805	Laser	~30	19 × 14	Yes

A successful device should be able to display RGB white light imaging, fluorescence imaging, and overlay imaging. The device should be capable of quantitating the light intensity to the extent possible. Quantitation permits FGS to be used in multicenter trials and allows comparison at different time points in the same patient. Further investigations are needed to establish reliable quantitative analyses of fluorescent imaging.

## Current Clinical use of Fluorescence Imaging Probes

Biomedical fluorescence imaging operates in wavelengths in the visible spectrum (400–700 nm), extending into the near infrared (NIR) spectrum (700–900 nm). A large number of commercially available fluorophores are available; however, few are clinically approved. While the majority of fluorescent probes emit light in the visible range, this is probably the least desirable part of the spectrum due to overlap with tissue autofluorescence and high absorbance of light in tissue in the visible spectrum. NIR fluorophores are better suited for *in vivo* imaging. While, wavelengths below 700 nm are strongly absorbed in tissue by endogenous molecules, such as hemoglobin and myoglobin, wavelengths above 900 nm are limited by water and lipid absorption wavelengths (153–155). Fluorophores emitting light <700 or >900 nm are, therefore, limited in their ability to penetrate tissue (156). The “NIR window” from 700 to 900 nm arises from less absorbance in tissues, allowing for deeper imaging and detection (153, 154). Thus, fluorophores in the NIR range have excellent potential for FGS. Fluorescence imaging using NIR fluorophores enhances cancer surgery navigation and offers higher sensitivity when compared to preoperative imaging, visual inspection, and palpation during surgery (157). Next, we will focus on the currently used fluorescence imaging probes in surgical oncology (Table 3).

**Table 3:** Currently used Food and Drug Administration-approved fluorescence probes.

Fluorescence probe	Excitation	Emission	Fluorescence type
Indocyanine green	780 nm	820 nm	Indocyanin green
Methylene blue (MB)	670 nm	690 nm	MB
5-Aminolevulinic acid (5-ALA)	380–440 nm	620 nm (alkaline pH) 634 nm (acid pH)	Porphyrin
Fluorescein sodium	494 nm	512 nm	Fluorescein
Folate	495 nm (folate-FITC)	520 nm (folate-FITC)	Fluorescein isothiocyanate (FITC)
IRDye800CW conjugate	775 nm	796 nm	IRDye800
IRDye700DX conjugate	680 nm	687 nm	IRDye700
Activatable probes	Various	Various	Various

### Indocyanine Green (ICG)

Currently, ICG is one of the most frequently employed NIR fluorophores used for FGS. ICG is a water-soluble, anionic, amphiphilic tricarbocyanine probe with a molecular weight of 776 Da (158, 159), which rapidly binds to plasma proteins in the body. The excitation peak is 780 nm and the emission peak is at 820 nm, which places outside the range of most tissue autofluorescence. ICG was first produced in 1955 by the Kodak Research Laboratories, and in 1959 it was approved by the FDA for retinal angiography. Historically, it has been clinically used to measure cardiac output (160), hepatic function (161), and retinal angiography (162).

Throughout its history, ICG has maintained a high safety index (25, 163, 164), as the number of allergic reactions is very low (1:10,000, as reported by manufacturer) (165). ICG also allows multiple repeated uses due to its short half-life of 150 to 180 s and is cleared exclusively by the liver (166).

Near infrared ICG-guided SNL mapping has been performed in various cancers as shown in Table 1. ICG has also been used for lymphography (167), angiography (61, 168), reconstructive surgery (65, 67), cholangiography (71) and tumor imaging (99), and so on. The use of ICG for delineating tumors has been a success. For instance, ICG fluorescence imaging identified 100% of primary hepatocellular carcinomas (HCCs) and in 40% of the cases also identified additional, small (3–6 mm) HCCs that would otherwise have gone undetected (98).

### Methylene Blue (MB)

Methylene blue is a heterocyclic aromatic compound with a molecular weight of 320 Da (51). It is a FDA-approved visible (dark blue) contrast agent. When sufficiently diluted, MB acts as a near-infrared fluorescent dye that operates within the NIR optical window with an absorption peak at 670 nm and an emission peak at 690 nm and is naturally excreted through the urine (51). MB was the first entirely synthetic drug used in medicine and was used in the treatment of malaria as early as 1891 by Guttmann and Ehrlich (169). MB continues to be applied and investigated as treatment for a variety of medical applications in the clinical setting, including methemoglobinemias and ifosfamide-induced encephalopathy (170, 171). MB has also been used to identify breast cancer (101) and neuroendocrine tumors (73), and is commonly used for SLN mapping (38, 39), as well as the identification of urologic tumors (51, 52) and tumors in the parathyroid glands (130).

Methylene blue is known to be relatively safe; however, the use of MB can potentially lead to cardiac arrhythmias, coronary vasoconstriction, decreased cardiac output, decreased renal blood flow and mesenteric blood flow, and increased pulmonary vascular pressure (172). Although MB accumulates in most tumors, the amount of accumulation varies with tumor type. Therefore, dye concentration appropriately matched to each tumor type is required (38).

### 5 -Aminolevulinic Acid (5-ALA)

5-ALA is the major substrate for protoporphyrin synthesis, and has been used clinically for tumor detection and tumor treatment (photodynamic therapy; PDT), as a FDA-approved substance. 5-ALA, typically administered in a topical or oral form, induces synthesis and accumulation of the fluorescent molecule protoporphyrin IX (PpIX) in epithelia and neoplastic tissues (83, 85, 86). 5-ALA-induced PpIX exhibits multiple physiochemical states depending on the microenvironment. One of the most important parameters affecting the state of PpIX is pH. In the pH range 3 to 11.5, there are two distinct states: emission peaks at 620 nm in alkaline environments and emission peaks at 634 nm in acidic environments after excitation with visible blue light of 380–440 nm (173, 174).

Cancer specific FGS with 5-ALA has been successfully implemented for resection of malignant gliomas in Europe after studies clearly demonstrated clinical benefits with regards to completeness of tumor removal (65% complete resection with 5-ALA compared to 36% in the white light group) and progression-free survival with its use (83). 5-ALA and derivatives have also been described in bladder cancer (117, 118, 120) and prostate cancer (122).

The use of 5-ALA has been limited by its relatively high costs, and an inconvenient method of administration (it is administered orally some hours before it is to be used). The high risk of skin sensitization within 24 h after the operation (the patient should not be exposed to sunlight or strong artificial light) also presents a challenge to its use (175).

### Fluorescein Sodium

Fluorescein sodium is a fluorescent drug that can be used intravenously to improve visualization of brain tumor tissue based primarily on non-specific vascular leakage. It is also used for retinal angiography (56–58). Fluorescein sodium is a sodium salt and an organic fluorescent dye with peak excitation at 494 nm and peak emission at 512 nm. It has been safely used in humans for many years, predominantly in ophthalmology for retinal angiography, and the cost of fluorescein sodium is relatively low when compared with the cost of 5-ALA (176). Fluorescein sodium is usually visible to the naked eye at high dosages (20 mg/kg body weight), and is observable through the yellow 560 nm filter at lower doses, allowing better tissue discrimination with more natural colors (177, 178).

The use of fluorescein sodium for the identification of intracranial tumors has been known since 1947 (179). As an FGS agent, fluorescein sodium has been commonly used for identifying glioblastoma (88) and metastatic brain tumors (92, 93). It has also been used for intracranial angiography (56–58).

## New Fluorescence Imaging Probes

The ideal fluorescence imaging probe must provide excellent contrast between the tumor or affected lymph node and healthy tissue (180). Therefore, a current challenge is to design fluorescent imaging probes with high selectivity for tumors, high tumor to background ratios, and minimal toxicity (155).

Current clinical studies are based on contrast agents that have already been approved. The most often used fluorophores are blood pool agents (including ICG) that have no inherent specificity for tumor or normal tissues, and thus are not ideal fluorophores for FGS. A number of new agents are currently being investigated, including several dyes from the cyanine family, such as Cy5.5, Cy7, Cy7.5, IR-dyes, nanoparticle formulations, and visible spectrum dyes (181). Most research focuses on increasing the availability of novel, fluorescently labeled agents to identify crucial landmarks, such as tumor margins, lymph nodes, and vital structures of interest to surgeons. A new generation of agents that target-specific antigens have been based on antibodies (113, 182, 183), nanobodies (184), aptamers, and peptides (77). Other approaches make use of enzymes for fluorescence activation (185–189).

In the following sections, we summarize progress made in several specific targeted optical imaging agents for FGS.

### Folate-Targeted FGS

The Folate receptor is commonly upregulated on tumor cells and, therefore, is a good candidate for a general-purpose fluorescently labeled, targeted agent. An example is folate fluorescein isothiocyanate (folate-FITC) that excites at a wavelength of 495 nm and emits at 520 nm (190). Folate and these folate analogs are internalized in the cell *via* receptor-mediated endocytosis within 2 h (191). Once inside the endosome, the conjugate remains intact and can, therefore, remain fluorescent after internalization (191, 192). This stability led to the development of a broad variety of folate-targeted conjugates. van Dam et al. used a folate fluorescein isothiocyanate to identify tumor implants in ovarian cancer patients who were undergoing abdominal surgery (28). Lung adenocarcinoma is also known to express high levels of folate receptor α (193, 194). This was exploited by Okusanya et al. who demonstrated that lung adenocarcinomas demonstrated fluorescence in 92% (46/50) of patients with folate-FITC (106). Another folate analog, EC17, was also used for imaging renal cell carcinoma, although only two of four cancers were detected (116). In another study using EC17 for intraoperative detection of ovarian cancer, Tummers et al. showed that the addition of FGS resulted in a16% increase in the resection of malignant tumors when compared to visual inspection and palpation (110). Of course, the clinical significance of this increase is still uncertain. Another folate analog, OTL38, has been used to delineate renal cell carcinoma margins during partial nephrectomy (102), and to identify ovarian cancer (111). Hoogstins et al. also reported that OTL38 accumulated in folate receptor α-positive tumors and metastases in 12 patients with ovarian cancer, enabling the surgeon to resect an additional 29% of malignant lesions that were not identified by inspection and/or palpation (111). Recently, both EC17 and OTL38 were also used for intraoperative lung tumor imaging (107, 108). Like all targeted agents, folate-FITC is restricted to use only in tumors over expressing folate receptor and, by virtue of the visible light emitted by FITC, the agent has a limited depth penetration.

### Monoclonal Antibody-Based Fluorescent Probes

Perhaps the most generalizable FGS probes are based on monoclonal antibodies (mAb) conjugated to a fluorescent dye. There are at least two scenarios in which mAb-based fluorescent probes could become clinically useful. One is in fluorescence-guided navigation to aid surgeons in detecting tiny lesions and determining the margin between cancer and normal tissue. Another is in selecting patients whose cancer cells express a sufficient amount of target to enable molecularly targeted therapies such as antibody–drug conjugates or antibody-photo-absorber conjugates.

Promising preclinical examples of targeted fluorescently labeled probes include anti carcinoembryonic antigen in pancreatic cancer and colorectal cancer conjugated to a green fluorophore (113), anti carbohydrate antigen 19-9 in pancreatic cancer conjugated to a green fluorophore (114), epidermal growth factor receptor (EGFR) and EGFR type2 (HER2) in breast cancer (195–197) and prostate-specific membrane antigen (PSMA) in prostate cancer conjugated to ICG (121). Most mAb-based fluorescent probes are designed for systemic administration. Compared to other routes of administration, such as oral or intra-tumoral injection, systemic administration allows for more homogeneous microdistribution. Moreover, systemic administration allows for sufficient washout time to elapse, to allow for the elimination of non-specific fluorescence from the blood and the urinary tract (22). In contrast to intra-tumoral injection, systemic administration also allows the detection of previously unrecognized tumor foci or metastases.

Few of these mAb-based fluorescent probes have progressed into clinical testing. One that did, a first-in-human clinical trial of fluorescence-guided navigation to aid surgery in head and neck cancers is currently underway. This trial utilizes the anti-EGFR antibody conjugate, cetuximab-IRDye800CW for use in head and neck cancers (94, 95). In this trial, Rosenthal et al. demonstrated that the EGFR mAb-fluorophore conjugate was both safe and effective. The target-to-background ratio (TBR) achieved in this study (mean TBR of 5.2 in the highest dose range) improved the accuracy of surgical decision-making (95). Recently, cetuximab-IRDye800CW or bevacizumab-IRDye800CW (targeting vascular endothelial growth factor)-have also been in clinical trials targeting pancreatic adenocarcinoma, colon cancer, and breast cancer (102, 126). It was noted that the conjugation of the IRDye800CW significantly shortened the circulating half-life of cetuximab despite a low antibody-to-dye conjugation ratio (approximately 1) (95). Rapid clearance of mAb–dye conjugates can help lower the background signal; however, it can simultaneously compromise tumor accumulation. Taken together, these alterations could lower the overall performance of the agent.

Another exciting advancement with potential implications for FGS is a technique called “near infrared photoimmunotherapy” (NIR-PIT) (96). NIR-PIT is based on an antibody that targets a cell-surface antigen but is conjugated to a photoabsorbing dye (IRDye700DX) that has both fluorescent characteristics and the ability to damage cells to which it has conjugated. Thus, NIR-PIT has the dual ability to localize tumors and as well as selectively eliminate cancer cells. The cytotoxic effects of NIR-PIT are observed only when the mAb-IR700 conjugate is bound to receptors on the cell membrane; no phototoxicity is observed when the conjugate is present but not yet bound (96). Therefore, NIR-PIT achieves highly selected targeted cancer cell killing. NIR-PIT has been shown to be effective in a variety of different cancer cell types exhibiting a range of surface antigens such as EGFR, CD20, mesothelin, and PSMA (198–202). Furthermore, a first-in-human phase 2 trial of NIR-PIT in patients with inoperable head and neck cancer was recently completed and the agent is being commercialized. NIR-PIT has great potential as a new cancer treatment for many tumor types when combined with FGS.

These fluorescence imaging probes, including IRDye700DX and IRDye800CW, typically result in an “always-on” type of fluorescence signal. Therefore, fluorescence in the cancer tissues is roughly related to the amount of conjugated mAb bound to the tumor. By showing sufficient expression of target molecules, activatable fluorescent probes would be useful for selecting eligible patients who could be efficiently treated with FGS.

### Activatable Fluorescent Probes

Based on pharmacokinetics, the “perfect” *in vivo* targeting agent has not yet been developed. The fundamental disadvantage of “always-on” probes is that they emit signal regardless of their proximity or interaction with the target tissues. As a result, there is considerable background signal to contend with. In order to design superior molecular imaging probes, one seeks to either (1) maximize signal from the target, (2) minimize signal from the background, or (3) do both. All lead to improved TBR, which, in turn, improves the sensitivity and specificity for detecting tumors with imaging (203).

**Figure 1:**
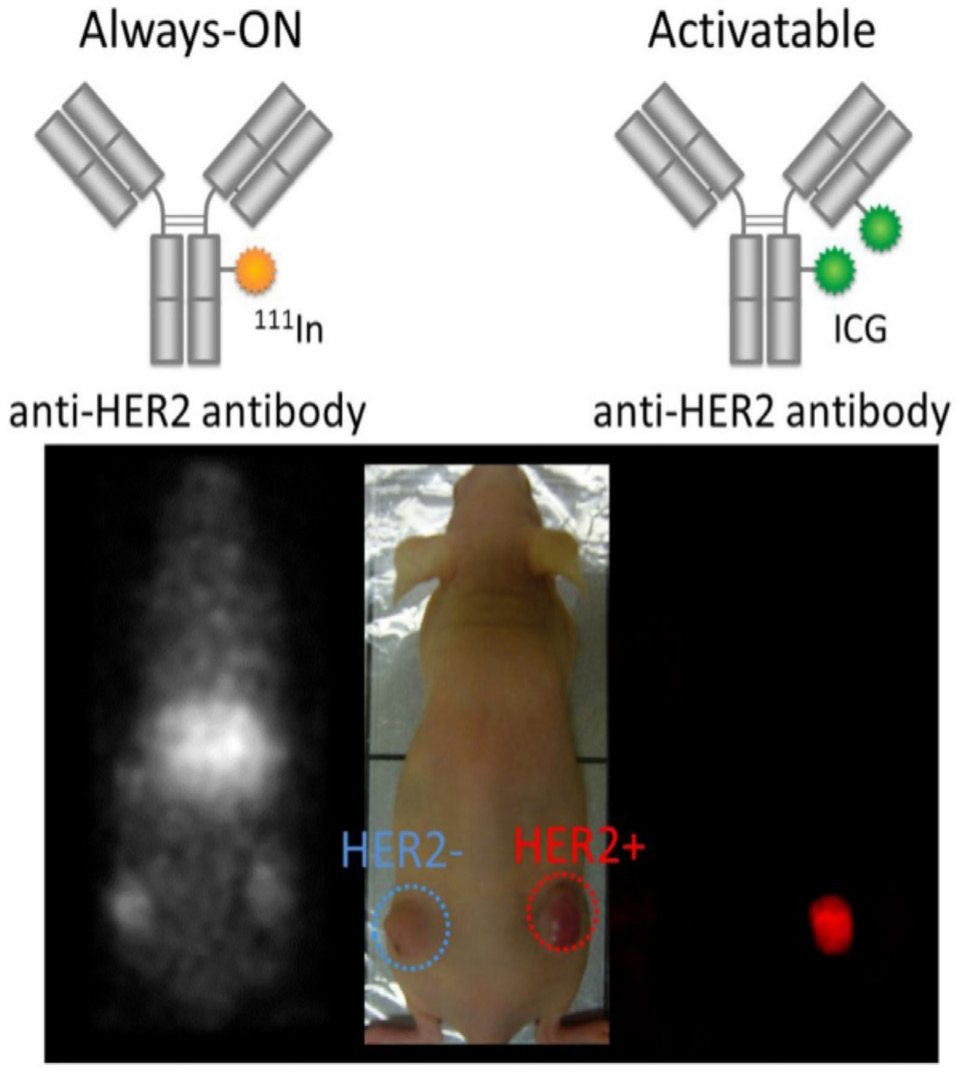
Comparison of molecularly targeted fluorescent probes using always-on and activatable fluorescence strategies. Radiolabeled trastuzumab targeting HER2 with always-on fluorophores depicts both bound and unbound agents (left and right tumors) resulting in poor target-to-background ratio (TBR). In contrast, the activatable fluorescent probe, indocyanine green (ICG)-labeled trastuzumab, depicts only HER2-expressing tumors (right tumor) without incurring background signal resulting in superior TBR. Reprinted from publication (210) with permission from Elsevier.

Activatable fluorescent probes (“smart probes”) target tumor cells by taking advantage of the physiologic differences between cancerous and normal cells, thus enhancing tumor margin detection (204). Because activatable probes do not emit signals before engaging the target, unbound probes do not yield a signal. Therefore, there is less background signal to compromise the sensitivity and specificity, yielding an absolute increase in TBR (205). Consequently, compared with “always-on” fluorescent probes, activatable fluorescent probes have a higher TBR (203). Preclinical studies have shown the merits of this approach (206–208). For example, when employing the antibody as a platform for activatable imaging probes, IgG-based activatable probes typically yield both the highest signal (due to high binding) as well as highest TBR (due to absent background signal) compared with “always-on” probes (203, 209, 210) (Figure 1).

**Figure 2:**
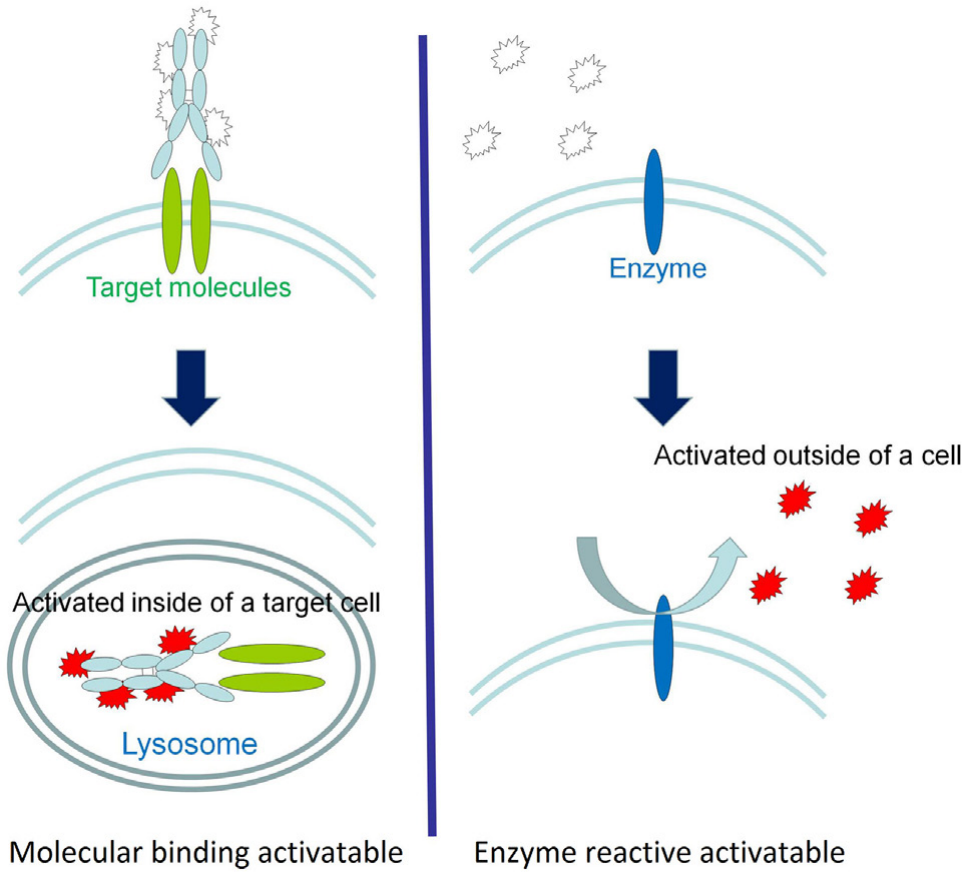
A schematic explanation of the two types of activatable fluorescence probes. The fluorescence activation of molecularly targeted activatable probes occurs intracellularly (left), whereas enzyme reactive activation typically occurs in the extracellular environment (right). Reprinted with permission from Ref. (203). Copyright 2011 American Chemical Society.

There are two basic types of activatable fluorescent probes (Figure 2) (203, 207, 211). One type is enzyme reactive activatable fluorescent probe, which exist in the quenched state until they are activated by enzymatic cleavage mostly outside of the cells (212, 213) (Figure 2). Well known targeted enzymes are cathespsin, matrix metalloproteinases (MMP), γ-glutamyltransferase (GGT), and beta-galactosidases. Some of enzyme reactive activatable fluorescent probes can be topically or locally applied.

**Figure 3:**
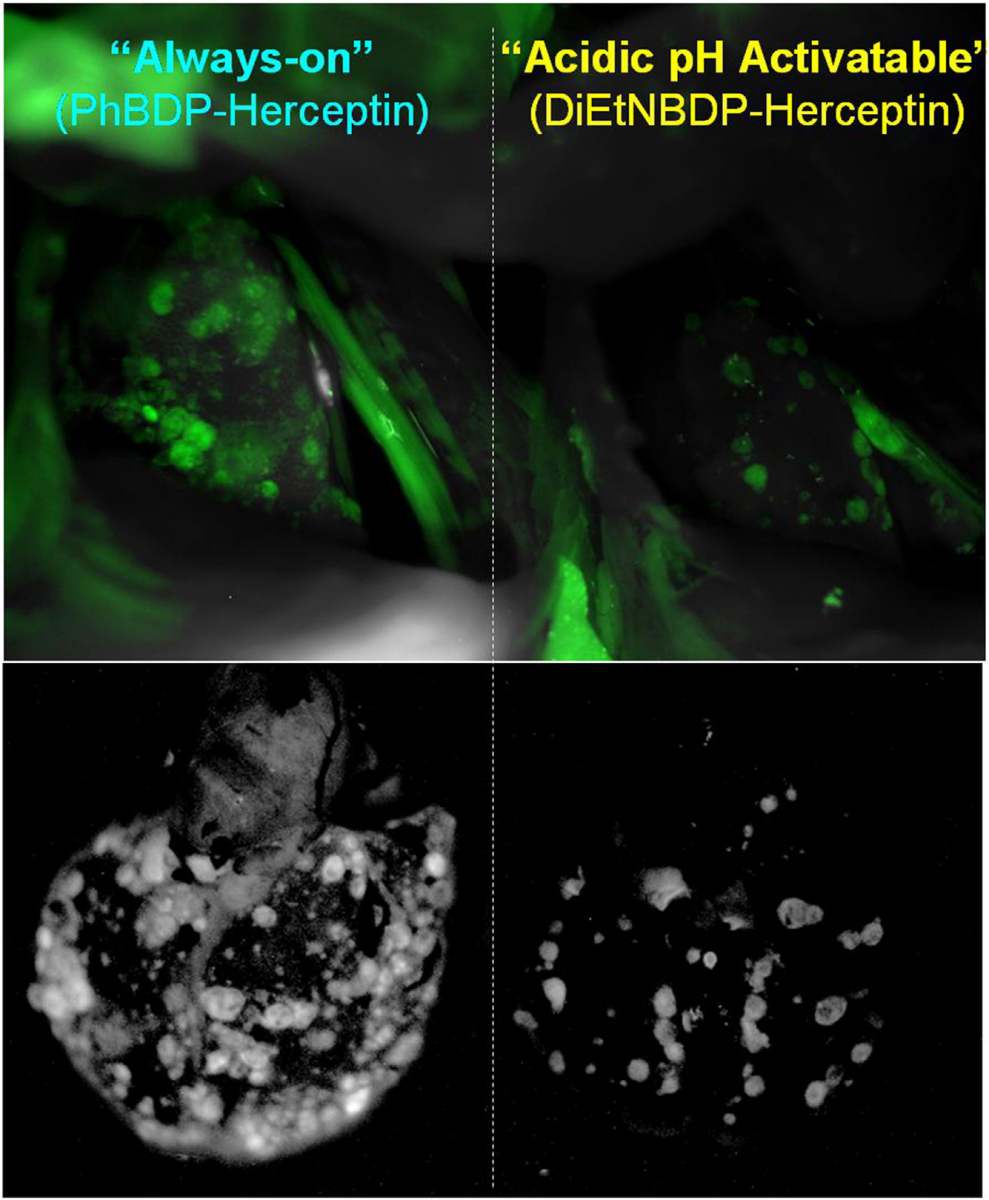
*In vivo* tumor detection with targeted activatable fluorescent probes in a HER2-positive lung metastasis model mice. The pH-activatable fluorescent probe produces light only in tumors in the lung. However, the control “always-on” probe produces fluorescent signal from both tumors and normal lung and heart reducing the tumor to background ratio. Reprinted with permission from Ref. (155). Copyright 2010 American Chemical Society.

Another type of activatable fluorescent probe is molecular-binding activatable fluorescent probes, which are quenched until activated in targeted cells by endolysosomal processing (Figure 2). Within the lysosome, catabolism can occur under conditions such as low pH, protease activity, or oxidation, which can release the fluorophore from its quenched state. For example, a pH-activatable fluorescent probe produces light only in tumors due to their acidic microenvironment, resulting in high TBR whereas control “always-on” probes produce lower TBR due to higher background signal (Figure 3) (155, 214). This type of activatable fluorescent probe is administered systematically *via* intravenous injection.

There are advantages and disadvantages to both methods. In enzymatic activation, a single target enzyme can activate many different fluorescent molecules, thus amplifying the signal from the target tissue. However, a disadvantage of enzymatic activation is that the activation occurs in the extracellular space and the enzyme may diffuse away from the target contributing to background signal. Furthermore, this type of probe lacks specificity because none of the currently utilized enzymes for fluorescence activation are specific for carcinogenesis. In contrast, probes that are activated by endolysosomal processing, are highly specific for cancer and generally remain localized to the target as activation relies upon the probe binding specific cell-surface receptors and being internalized. However, molecular-binding of specific activatable fluorescent probes requires a biological and catabolic process to gain sufficient TBR. Targeted activatable fluorescent probes need to first leak from the vasculature, bind target cells, and then internalize within the target cell to activate the probe. The activation process often requires days, which decreases their practicality for routine clinical use (214). Novel activatable fluorescent probes targeting additional physiologic characteristics of cancer cells, such as degradation of the micelle, thiol concentration, surface lectins, and antibody binding, are also currently in development (159, 207, 211, 215–218). Translating these activatable fluorescent probes into clinical studies could significantly increase the number and quality of intraoperative imaging tools available during cancer excision.

Activatable fluorescent probes vary greatly by the mechanism by which fluorescence is quenched. The best known quenching mechanism is Föster (fluorescence) resonance energy transfer (FRET), wherein energy from one fluorophore is transferred to another molecule when the two molecules are in close (<10 nm) proximity. The FRET pair can consist of two fluorophores (self-quenching) or a fluorophore and a quencher molecule (203, 219). Homo-dimer (H or J-dimer) formation is other method of quenching. For instance, xanthene derivatives are known to form H-dimers at higher concentrations (~mM) which induces shifts of absorbance spectra, completely quenching fluorescence (195, 203). Fundamental to both FRET and H-dimer formation is the inter-fluorophore processing that occurs when the two molecules are in proximity to each other.

Another quenching mechanism, photon induced electron transfer (PeT) occurs when an electron is transfered from the PeT donor to the excited fluorophore diminishing the fluorescence signal. When the PeT donor is cleaved from the fluorophore or inactivated, activation occurs. Unlike FRET and H-dimer formation, PeT occurs within a single fluorophore molecule and does not require the presence of a second fluorophore (203, 220). The PeT mechanism has a particularly high dequench:quench light ratio.

Yet another mechanism of dequenching is to hold two fluorophores in close proximity to each other using a peptide backbone. In the presence of an enzyme the peptide backbone is cleaved releasing and activating the fluorophores.

The first activatable fluorescent probe to be tested in clinical trials was LUM015. The activation of LUM015 relied on cleavage by a cathepsin protease, an enzyme commonly overexpressed by tumors (221). LUM015 is optically inactive under normal conditions, but upon proteolytic cleavage, a covalently attached quencher molecule is released and fluorescence signal greatly intensifies (103). It was first evaluated by Whitley et al. in a cohort of 15 patients with breast cancer or soft-tissue sarcoma (103). Intravenous injection of this protease-activated fluorescent imaging probe before surgery was well tolerated, and imaging of resected human tissues showed that fluorescence from the tumor was significantly higher than fluorescence from normal tissues (103).

Recently, several other mechanisms have been tested. A quenched activatable cell-penetrating peptide, AVB-620 was tested in a first-in-human clinical trial in which 27 breast cancer patients received the infusion followed by surgical excision. Infusion of AVB-620 was safe and improved intraoperative cancer detection (104). Another new approach is to use peptide conjugated to ICG. In this case the agent, BLZ-100, uses chlorotoxin (36-amino acid peptide) as the targeting moiety and conjugates it to ICG. This agent has been used for glioma imaging (90).

### Sprayable Activatable Fluorescent Probes

**Figure 4:**
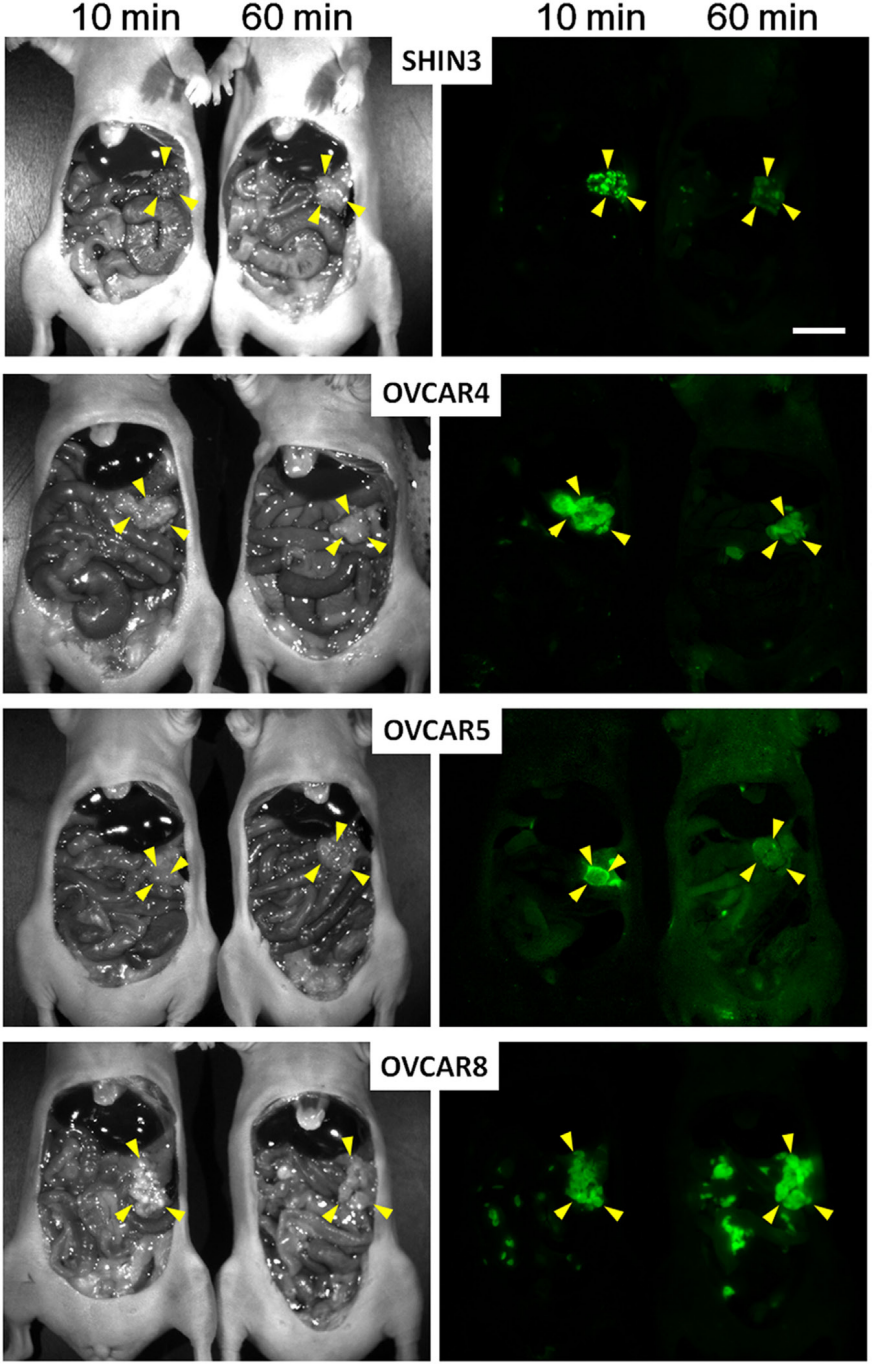
Spectral fluorescence images of four peritoneal ovarian cancers using gGlu-HMRG. *In vivo* fluorescence intensity of a sprayable probe. By 10 and 60 min after intraperitoneal gGlu-HMRG administration each of four peritoneal ovarian tumor models: SHIN3, OVCAR4, OVCAR5, and OVCAR8 were evaluated. Yellow arrowheads indicate tumor location. Scale bar, 1 cm. Reprinted from publication (112) with permission from AAAS.

In many cases, the dequenching process takes hours to days making it problematic for integration into surgical workflows. For instance, activatable probes using cathepsin D and MMP2/9 (222, 223), should be systemically injected at least a day before the surgery to be delivered to cancer and fully activated because of multiple cleavage sites. However, the kinetics of some other enzyme reactive probes is much faster especially when activated by a single cleavage. Therefore, such enzymatically activatable fluorescent probes can be so fast as to be used as needed during a surgical procedure. For instance, Urano et al. developed the activatable fluorescent probe, γ-glutamyl hydroxymethyl rhodamine green (gGlu-HMRG). The gGlu-HMRG is completely quenched by spirocyclic caging, but is activated rapidly with a one-step enzymatic reaction in the presence of GGT which is often present on cell membranes of cervical and ovarian cancer cells. As a result, this probe activates within 10 min of it being sprayed on. In a mouse model of human ovarian cancer, Urano et al. sprayed the abdominal cavity with the gGlu-HMRG probe and demonstrated that small tumor nodules could be visualized within 10 min after administration and remained labeled for at least 1 h (Figure 4) (112). Mitsunaga et al. used gGlu-HMRG during colonoscopy to differentiate long-term colitis from early colitis-associated cancer in a mouse colon cancer mouse model. They were able to visualize cancers and dysplasia 5–30 min after spraying gGlu-HMRG on the colon surface. Moreover, signal from cancer/dysplasia was 10 times higher than background fluorescence despite the presence of colitis (127). gGlu-HMRG probe has recently been tested in fresh human surgical specimens of colorectal tumor (224) and breast cancer (225) for detecting tumor borders and metastatic lymph nodes as a precursor to it being introduced in clinical trials. Similarly, these probes revealed that topical administration of the agent on aspirated specimens from patients with pancreatic tumors resulted in tumor-specific enhancement (226).

Other sprayable activatable probes are in development. These are activated by enzymes by a single cleavage such as cathepsin (91, 129), beta-galactosidase (227), endo-aminopeptidases (228), and NADPH (229).

The various types of recently developed activatable fluorescent probes tend to be superior to always-on probes; however, their safety in patients is yet to be determined. Given the relatively small market, bringing such agents thru the approval process to a New Drug Application will be challenging.

## Summary

The limits of white light imaging during surgical and endoscopic procedures are well known. It is acknowledged that current optical methods tend to have insufficient sensitivity for small tumors and do poorly at determining tumor margins. Targeted fluorescence imaging can provide additional information that augments the ability of the operator to see and treat pathology, thus lowering the rate of persistent or recurrent disease. FGS, because of its high sensitivity, low cost, portability and real-time capabilities, has great potential to improve surgical outcomes. Not only can this approach direct intraoperative image guidance for surgical margin assessments but can also help surgeons detect microscopic tumors or residual lesions that may have otherwise been missed. In addition, anatomical fluorescence imaging techniques can aid in avoiding complications in various surgical situations. Despite the availability of these technologies, most surgeons still rely largely on visual and tactile cues combined with presurgical radiologic imaging to guide tissue resection.

As techniques continue to improve, FGS will move toward the concept of “precision surgical therapy.” It is possible that FGS will be personally designed for each patient’s specific disease process. Although much more work is necessary to reach this goal, in the meantime there is a rapidly expanding number of targeted fluorescence imaging probes that offer great potential for the future. Hopefully, these advances will enable FGS to become more widely available for a broad range of cancer types.

## Author Contributions

All authors listed have made a substantial, direct, and intellectual contribution to the work and approved it for publication.

## Conflict of Interest Statement

The authors declare that the research was conducted in the absence of any commercial or financial relationships that could be construed as a potential conflict of interest.

## Abbreviations

AAA, abdominal aortic aneurysm; ALA, aminolevulinic acid; ADC, antibody-drug-conjugate; APC, antibody-photo-absorber conjugate; CA, carbohydrate antigen; CABG, coronary artery bypass grafting; CEA, carcinoembryonic antigen; CT, computerized tomography; epidermal growth factor receptor; FDA, Food and Drug Administration; FGS, fluorescence-guided surgery; FITC, fluorescein isothiocyanate; FLARE, fluorescence-assisted resection and exploration; FRET, Föster (fluorescence) resonance energy transfer; gGlu, γ-glutamyl; GGT, γ-glutamyltransferase; H-dimer, homo-dimers; HLA, hexaminolevulinate; HMRG, hydroxymethyl rhodamine green; ICG, indocyanine green; mAb, monoclonal antibodies; MB, methylene blue; MMP, matrix metalloproteinase; MRI, magnetic resonance imaging; NDA, New Drug Application, NIR, near-infrared; PET, positron emission tomography; PeT, photon induced electron transfer; PIT, photoimmunotherapy; Pp, protoporphyrin; PSMA, prostate-specific membrane antigen; SLN, sentinel lymph node; TBR, target-to-background ratio; VEGF, vascular endothelial growth factor.
